# Curcumin attenuates lupus nephritis by inhibiting neutrophil migration via PI3K/AKT/NF-κB signalling pathway

**DOI:** 10.1136/lupus-2024-001220

**Published:** 2024-07-24

**Authors:** Hui Yang, Haiwei Zhang, Lili Tian, Panpan Guo, Shanshan Liu, Hongwei Chen, Lingyun Sun

**Affiliations:** 1 Department of Rheumatology and Immunology, Nanjing Drum Tower Hospital Clinical College of Nanjing University of Chinese Medicine, Nanjing, Jiangsu, China; 2 Department of Rheumatology and Immunology, Nanjing Drum Tower Hospital Clinical College of Xuzhou Medical University, Nanjing, Jiangsu, China; 3 Department of Rheumatology and Immunology, Nanjing Drum Tower Hospital, Affiliated Hospital of Medical School, Nanjing University, Nanjing, Jiangsu, China

**Keywords:** lupus nephritis, autoimmune diseases, cytokines, inflammation, lupus erythematosus, systemic

## Abstract

**Objective:**

To investigate the role of curcumin in the treatment of lupus nephritis (LN) by inhibiting the migration of neutrophils and the underlying mechanism involved.

**Methods:**

Two lupus mouse models, MRL/lpr mice and R848-treated mice, were treated with 50 mg/kg curcumin by intraperitoneal injection. H&E and Masson staining were used to estimate histopathological changes in the kidney. Immunofluorescence was used to assess the deposition of immune complexes. The expression of inflammatory factors was detected by enzyme-linked immunosorbent assay (ELISA) and real-time reverse transcription polymerase reaction (RT-PCR), and the protein expression was detected by western blotting.

**Results:**

We revealed the remarkable potential of curcumin in improving inflammatory conditions in both MRL/lpr mice and R848-induced lupus mice. Curcumin effectively decelerates the progression of inflammation and diminishes the infiltration of neutrophils and their release of pivotal inflammatory factors, thereby reducing inflammation in renal tissues. Mechanistically, curcumin significantly inhibits the expression of p-PI3K, p-AKT and p-NF-κB, which are upregulated by interleukin-8 to induce neutrophil migration and renal inflammation, thereby reducing neutrophil migration and the release of inflammatory factors.

**Conclusion:**

Curcumin significantly inhibits the recruitment of neutrophils and the release of proinflammatory factors in the kidney by inhibiting the PI3K/AKT/NF-κB signalling pathway, providing new therapeutic targets and medication strategies for the treatment of LN.

**WHAT IS ALREADY KNOWN ON THIS TOPIC**

- Curcumin is known for its anti-inflammatory properties, and neutrophil migration is crucial in lupus nephritis; however, the effect of curcumin on neutrophil migration in lupus nephritis remains unclear.

**WHAT THIS STUDY ADDS**

- Curcumin alleviates renal pathological damage in lupus mice by inhibiting neutrophil infiltration and migration through the regulation of the phosphatidylinositol 3-kinase/AKT/nuclear factor-κB signalling pathway.

**HOW THIS STUDY MIGHT AFFECT RESEARCH, PRACTICE OR POLICY**

- This study suggests that curcumin can be considered as a potential therapeutic agent for treating lupus nephritis in clinical practice, particularly in targeting neutrophil-driven inflammation.

## Introduction

 Systemic lupus erythematosus (SLE), a chronic autoimmune disease, is characterised by autoantibody, immune complex and organ dysfunction.^1^ Lupus nephritis (LN) is a common complication of SLE, as approximately 60% of patients have clinical manifestations of renal impairment, which is the main cause of mortality in SLE patients.^2^,^4^ The pathogenesis of LN encompasses intricate mechanisms wherein dysregulated activation of both innate and adaptive immunity triggers the activation and proliferation of various immune cell populations, such as monocytes, autoreactive B cells and T cells. These cells are recruited to the kidney and exhibit a heightened propensity to generate copious amounts of detrimental cytokines and chemokines, attracting more inflammatory cells in turn.^5^,^7^ Currently, immunosuppressants, such as mycophenolate mofetil, cyclophosphamide and glucocorticoids, are typically used to treat LN.^8^ However, the effectiveness of these drugs is inconsistent because 35% of patients may relapse after taking immunosuppressive agents.^9^,^11^ In addition, long-term and high-dose use of immunosuppressants results in toxic side effects, significantly compromising the survival and quality of life of patients.^12^ Therefore, it is urgent to find an alternative approach that can effectively improve LN with fewer adverse reactions.

Infiltration of immune cells is a major sign of LN. Moreover, renal biopsies from patients with LN have shown that active LN is associated with infiltrated neutrophils.^13^,^15^ Previous studies have shown that the inhibition of neutrophil recruitment and subsequent release of inflammatory cytokines in the renal milieu not only serve to prevent neutrophil-mediated impairment of target organ functionality but also exert suppressive effects on intrarenal autoimmune responses.^16^ Therefore, the inhibition of neutrophil recruitment in the kidney seems to be a potential target for the treatment of LN.

Curcumin, a safe natural diketone compound extracted from the rhizome of turmeric, has shown excellent anti-inflammatory, antioxidative and antitumour properties.^17^,^19^ It has been extensively used to treat cancer, inflammatory bowel disease, metabolic disease, autoimmune diseases and infectious diseases.^20^,^23^ Curcumin improves autoimmune disease by inhibiting the release of inflammatory cytokines (tumour necrosis factor (TNF)-α, interleukin (IL)-1β, IL-6, interferon (IFN)-γ, etc), as well as the related nuclear factor-κB (NF-κB), AKT or phosphatidylinositol 3-kinase (PI3K) signalling pathways in immune cells.^24^,^26^ In addition, this natural compound ameliorates organ damage in immune diseases by inhibiting the recruitment of immune cells to inflammatory sites and the subsequent release of inflammatory cytokines.^27^,^29^ Previous studies have shown that curcumin improves renal function in lupus mice by inhibiting the NLRP3 inflammasome.^30^ However, the mechanism by which curcumin alleviates LN through regulation of neutrophils remains unclear.

In this study, we explored the ability of curcumin to attenuate LN in two different mouse models. We further demonstrated that curcumin ameliorated LN by reducing neutrophil migration and inhibiting the secretion of inflammatory factors by regulating the PI3K/AKT/NF-κB signalling pathway. This study provides a new target and strategy for the treatment of LN with curcumin.

## Materials and methods

### Patients

All blood samples were obtained from inpatients in the Department of Rheumatology and Immunology of Nanjing Drum Tower Hospital, and all patients met the 1997 American College of Rheumatology criteria for SLE. We excluded patients with other types of autoimmunity, including diabetes, rheumatoid arthritis, Sjögren’s syndrome and systemic sclerosis. Healthy controls (HCs) were recruited from the Medical Examination Center of Nanjing Drum Tower Hospital. Informed consent was obtained from all patients and HCs for this study, and the information is shown in table 1.

**Table 1:** Clinical characteristics and medication for patients with LN

Patient	Age/Sex	Disease duration (months)	Anti-dsDNA	Scr (μmol/L)	24-hour urine protein (mg)	Medication
1	57/F	24	+	256	710	CTX, MMF, Pred
2	46/F	4	+	88	3441	MMF, HCQ
3	34/F	7	+	296.2	7127.5	MMF
4	20/F	6	+	78	4307	Pred, HCQ
5	59/F	10	+	146	5002	Belimumab

Anti-dsDNAantidouble-stranded DNACTXcyclophosphamideHCQhydroxychloroquineMMFmycophenolate mofetilPredprednisoneScrserum creatinine

### Isolation of neutrophils

Neutrophils were isolated from the peripheral blood of patients with SLE and HCs by using PolymorphPrep (TBD, Tianjin, China) according to the manufacturer’s instructions. Neutrophils were resuspended in RPMI 1640 (Gibco) supplemented with 1% penicillin-streptomycin (Gibco) and 10% fetal bovine serum (FBS, Gibco). A total of 1×10^6^ cells were obtained and incubated with 10 μM curcumin (HY-N0005, MedChemExpress, New Jersey, USA) at 37°C with 5% CO_2_ for 24 hours, after which the cells were collected for further experiments.

### Animals

Female MRL/lpr mice (aged 15 weeks) were purchased from SPF Biotechnology (Beijing, China). Female C57BL/6J mice were obtained from Huachuang Sino (Jiangsu, China). After 1 week of acclimatisation, the MRL/lpr mice (n=12) were intraperitoneally administered either vehicle (n=6) or curcumin dissolved in dimethylsulfoxide (n=6, 50 mg/kg) for 2 weeks. All mice were euthanised at 18 weeks of age. C57BL/6J mice (n=20) were treated with 50 μg of the TLR7 agonist resiquimod (R848; MedChemExpress) dissolved in 25 μL of acetone via application to the epidermis of the ear 3 times a week until euthanasia, and acetone alone was used as a control. During treatment with R848, the mice received vehicle (n=8) or curcumin (n=8, 50 mg/kg) daily by intraperitoneal injection.

### Flow cytometry

Peripheral blood mononuclear cells (PBMCs) were isolated from the peripheral blood of MRL/lpr and C57BL/6J mice using Mouse 1×Lymphocyte Separation Medium (TBD LTS1092, Tianjin, China). The spleen and perinephric lymph nodes of the mice were ground in phosphate-buffered saline (PBS) and filtered through a 200-mesh screen to prepare a single-cell suspension. A total of 1×10^6^ cells were obtained from mouse spleenocytes, lymphocytes or PBMCs and incubated with the following antibodies for 30 min in the dark at 4°C: L/D-APC-Cy7 (65-0865-18, Invitrogen), CD4-FITC (100406, BioLegend), CD8-BV510 (563068, BD) and CD25-APC (101909, BioLegend). Then, the cells were fixed and permeabilised with a fixation/permeabilisation kit (2367477, Invitrogen) for 1 hour in the dark at 4°C. The cells were washed with PBS and incubated with Foxp3-PE (126403, BioLegend) for 30 min in the dark at 4°C to evaluate the number of mouse T regulatory cells (Treg) cells. To make renal cell suspensions, kidneys were cut into small pieces, treated with 0.1% collagenase II (V900892, Sigma) and incubated at 37°C for 30 min for digestion. Then, 100 μm filters were used to filter the mixture to collect single cells. A total of 1×10^6^ cells were then incubated with the following antibodies at 4°C in the dark for 30 min: L/D-APC-Cy7 (65-0865-18, Invitrogen), CD45-PE Cy7 (103114, BioLegend), CD11b-BV421 (101236, BioLegend) and Ly6G-FITC (127606, BioLegend). The prepared cells were analysed by flow cytometry (BD LSRFortessa), and the data were processed via FlowJo software.

### Histological examination

Murine kidneys were fixed in 4% paraformaldehyde (PFA) for 24 hours. Then, the tissue was embedded in paraffin and continuously sectioned at 5 μm. The tissue sections were stained with H&E and Masson’s trichrome. The histological scores of the renal lesions were evaluated as described previously.^31^

### Immunohistochemistry

The tissue sections were dewaxed with xylene solution and then treated with citrate buffer (pH 6.0) at 120°C for 15 min. Subsequently, 3% hydrogen peroxide was used to inhibit the activity of endogenous peroxidases. After blocking with 5% bovine serum albumin (BSA) for 30 min, the slices were stained with Ly6G (polyclonal rabbit, 1:200, ab238132, Abcam) overnight at 4°C. Then, the slices were washed and incubated with horseradish peroxidase (HRP)-conjugated goat antirabbit IgG (1:200, GB23303, Servicebio) for 1 hour. The slices were sealed after restaining with methyl green (0.2%), and images were acquired using a microscope (Olympus IX73, Tokyo, Japan).

### Immunofluorescence

The kidney was embedded in optimal cutting temperature compound and continuously sectioned at 10 μm. Then, the slices were fixed with 4% PFA for 30 min, and after washing in PBS, the samples were treated with 1% H_2_O_2_ for 5 min. Next, the sections were blocked with 1% BSA for 30 min before staining with C3 (polyclonal rabbit, 1:1000, ab200999, Abcam) for 1 hour. Then, the slices were washed with PBS and incubated with secondary antibodies (goat antimouse IgG (H+L), 1:1000, ab150113, Abcam) and goat antirabbit IgG (H+L), 1:1000, 4413S, Cell Signaling Technology). Finally, the nuclei were visualised with 5 μg/mL Hoechst 33 258 (94403, Sigma) followed by slide coverings. The samples were imaged by using laser confocal scanning microscopy (Olympus FV3000, Tokyo, Japan).

### Transwell migration assay

Migration assays were carried out in a Transwell chamber with an aperture of 3 μm (14322, LabSelect, China). A total of 10^6^ HL60 cells (CL-0110, Pricella) were seeded into the chambers, and the chambers were placed in a 24-well plate containing 600 μL of Iscove’s Modified Dulbecco’s Medium (IMDM) containing 10% FBS and 1% penicillin-streptomycin (Gibco). Moreover, the medium was supplemented with or without IL-8 (100 ng/mL, HY-P7224, MedChemExpress) and curcumin (10 μM). After incubation for 2 hours, the chambers were washed with PBS and fixed with 4% PFA for 10 min. Crystal violet was used to stain the cells for 10 min, after which the cells were observed under a microscope (Olympus FV3000, Tokyo, Japan). Cells were counted in five random microscopic fields from each group.

### Western blot analysis

Protein extraction was performed by adding RIPA lysis buffer (Biosharp, China) containing 1 mM protein phosphatase inhibitor to the neutrophils. Then, the samples were centrifuged at 12 000 rpm at 4°C for 15 min to obtain the supernatants, and the protein concentration was determined by a BCA protein assay kit (Thermo Scientific). Proteins were separated on 10% sodium dodecyl sulfate-polyacrylamide gels and then transferred to PVDF membranes (Bio-Rad, Hercules, California, USA). After blocking with 5% skim milk at room temperature for 1 hour, the membranes were incubated with phosphorylated (p)-PI3K (1:1000, 4228T, Cell Signaling Technology), PI3K (1:1000, 4249T, Cell Signaling Technology), p-AKT (1:1000, 4060T, Cell Signaling Technology), AKT (1:1000, 4691T, Cell Signaling Technology), p-NF-κB (1:1000, 3033T, Cell Signaling Technology), NF-κB (1:1000, 8242T, Cell Signaling Technology) and GAPDH (1:1000, 5174T, Cell Signaling Technology) at 4°C overnight. Next, the membranes were washed with Tris-Buffered Saline Tween-20 (TBST) three times and incubated with secondary antibody (goat antirabbit IgG (H+L)-HRP, 1:5000, AFSA004, AiFang Biological) at room temperature for 1 hour. Chemiluminescence was performed using a ChemiDocXRS+Imaging System (Tanon, Shanghai, China). Quantitative analysis of proteins was performed by using ImageJ.

### Real-time RT-PCR

Total RNA was collected using TRIzol reagent (Vazyme, China), and the concentrations of RNA were determined by Nanodrop. Next, the PrimeScript RT reagent kit (Vazyme, China) was used to transcribe the RNA to complementary DNA. The PCR was performed on a QuantStudio 6 Flex (Applied Biosystems, Foster City, USA) by using SYBR Green I (Takara Biotechnology, Tokyo, Japan). The primer sequences are shown in table 2.

**Table 2:** Gene primers used in this study

Name	Primer list
*h-GAPDH*-F	5’-GTCTCCTCTGACTTCAACAGCG-3’
*h-GAPDH*-R	5’-ACCACCCTGTTGCTGTAGCCAA-3’
*h-TNFα*-F	5’-CTCTTCTGCCTGCTGCACTTTG-3’
*h-TNFα*-R	5’-ATGGGCTACAGGCTTGTCACTC-3’
*h-IL6*-F	5’-AGACAGCCACTCACCTCTTCAG-3’
*h-IL6*-R	5’-TTCTGCCAGTGCCTCTTTGCTG-3’
*h-IL1β*-F	5’-CCACAGACCTTCCAGGAGAATG-3’
*h-IL1β*-R	5’-GTGCAGTTCAGTGATCGTACAGG-3’
*h-IL8*-F	5’-GAGAGTGATTGAGAGTGGACCAC-3’
*h-IL8*-R	5’-CACAACCCTCTGCACCCAGTTT-3’
*h-IFNα*-F	5’-AGAAGGCTCCAGCCATCTCTGT-3’
*h-IFNα*-R	5’-TGCTGGTAGAGTTCGGTGCAGA-3’
*h-IFNβ*-F	5’-ATGACCAACAAGTGTCTCCTCC-3’
*h-IFNβ*-R	5’-GGAATCCAAGCAAGTTGTAGCTC-3’
*h-IFNγ*-F	5’-GAGTGTGGAGACCATCAAGGAAG-3’
*h-IFNγ*-R	5’-TGCTTTGCGTTGGACATTCAAGTC-3’
*m-Mrp8*-F	5’-CAAGGAAATCACCATGCCCTCTA-3’
*m-Mrp8*-R	5’-ACCATCGCAAGGAACTCCTCGA-3’
*m-Mrp14*-F	5’-TGGTGGAAGCACAGTTGGCAAC-3’
*m-Mrp14*-R	5’-CAGCATCATACACTCCTCAAAGC-3’
*m-Tnfα*-F	5’-GGTGCCTATGTCTCAGCCTCTT-3’
*m-Tnfα*-R	5’-GCCATAGAACTGATGAGAGGGAG-3’
*m-Il-1β*-F	5’-TGGACCTTCCAGGATGAGGACA-3’
*m-Il-1β*-R	5’-GTTCATCTCGGAGCCTGTAGTG-3’
*m-Ifn-γ*-F	5’-ATGAACGCTACACACTGCATC-3’
*m-Ifn-γ*-R	5’-CCATCCTTTTGCCAGTTCCTC-3’
*m-Tgf-β*-F	5’-TGATACGCCTGAGTGGCTGTCT-3’
*m-Tgf-β*-R	5’-CACAAGAGCAGTGAGCGCTGAA-3’
*m-α-Sma*-F	5’-CGGTACAGTACCGAATGCACCA-3’
*m-α-Sma*-R	5’-TGTCTCCTCTGTGTCAGTCACG-3’
*m-Gapdh*-F	5’-CATCACTGCCACCCAGAAGACTG-3’
*m-Gapdh*-R	5’-ATGCCAGTGAGCTTCCCGTTCAG-3’

### Enzyme-linked immunosorbent assay

The serum levels of IL-6 and IFN-γ in the mice were detected by ELISA kits (EK206 and EK280, Multi Sciences, China) according to the manufacturer’s instructions.

### Statistical analysis

All experiments were performed with three biological replicates, and the statistical analysis was performed with GraphPad Prism V.9.0 software (GraphPad, San Diego, California, USA). The statistical significance of differences between two groups was analysed by t-tests, and the statistical significance of differences among multiple groups was analysed by one-way analysis of variance with Tukey’s multiple comparisons test. The data are shown as the mean±SEM. P value <0.05 was considered to be statistically significant.

## Results

### Curcumin ameliorates lupus nephritis in MRL/lpr mice

**Figure 1:**
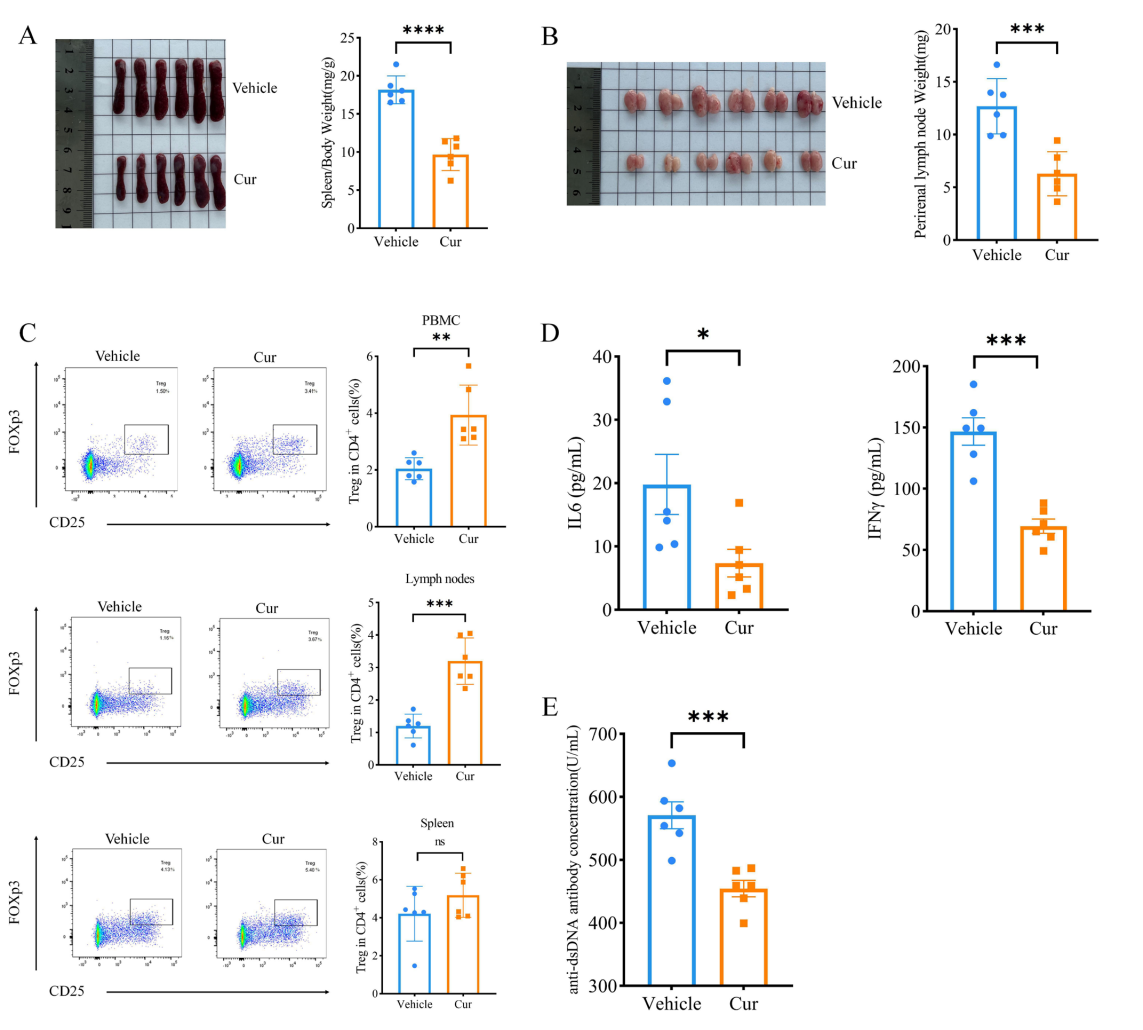
The effects of curcumin treatment on MRL/lpr mice. (**A**) Image of spleens and spleen/body weight ratios (mg/g) from the curcumin treatment (Cur) and control (Vehicle) groups. (**B**) Image of perirenal lymph nodes and the ratios of perirenal lymph nodes to body weight in the treatment and control groups. (**C**) Flow cytometric analysis of Treg cells in the PBMCs, lymph nodes and spleens of MRL/lpr mice treated with or without curcumin. (**D**) Serum IL6 and IFNγ levels measured by ELISA in the curcumin treatment and vehicle groups. (**E**) The serum level of anti-dsDNA antibody in curcumin and vehicle groups. The data are presented as the mean±SEM (n=6). ns, p˃0.05. *P<0.05, **p<0.01, ***p<0.001, ****p<0.0001. ds, double-stranded; IFN, interferon; IL, interleukin; PBMC, peripheral blood mononuclear cell.

To evaluate the therapeutic effect of curcumin on SLE, 16-week-old MRL/lpr mice were intraperitoneally injected with curcumin or vehicle daily for 2 weeks. Splenomegaly and lymph node enlargement are significant signs of lupus disease in MRL/lpr mice. Treatment with curcumin dramatically inhibited the enlargement of the spleen in MRL/lpr mice (figure 1A, p<0.001), reducing the weight of the perirenal lymph nodes (figure 1B, p<0.0001). As CD4^+^CD25^+^FoxP3^+^ Treg cell deficiency is considered to be involved in the pathogenesis of SLE,^32 33^ we next measured whether curcumin treatment could promote Treg cells to reinforce autoimmune tolerance. Our flow cytometry results showed that treatment of lupus mice with curcumin markedly increased the percentage of Treg cells among CD4^+^ cells in PBMCs and lymphocytes but that there was a trend toward increased Treg cells in the spleen, but the difference was not significant (figure 1C). Moreover, we measured the serum levels of the anti-dsDNA antibody, IL-6 and IFN-γ, which are also hallmarks of lupus progression. As expected, the ELISA results showed that the concentrations of the ds-DNA antibody, IL-6 and IFN-γ were notably lower in the curcumin treatment group than in the control group (figure 1D–E). Taken together, these results indicated that curcumin has a curative effect on MRL/lpr mice.

### Curcumin improves renal function in MRL/lpr mice

**Figure 2:**
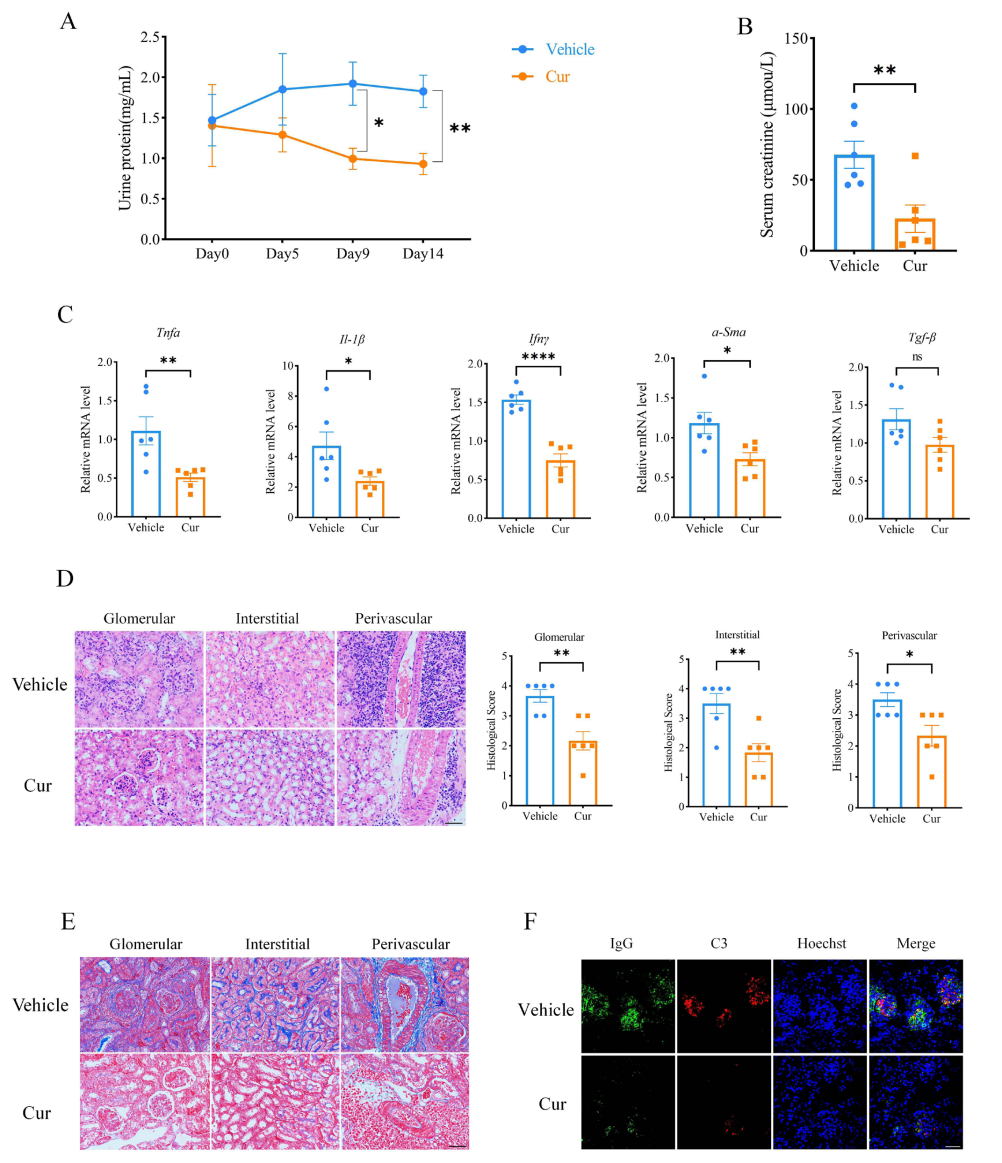
Curcumin improved renal function in MRL/lpr mice. (**A**) Measurement of urine protein in MRL/lpr mice from the curcumin treatment group and control group. (**B**) Serum creatinine levels measured by ELISA in the curcumin treatment and control groups. (**C**) Transcriptional expression of *Tnfα, Ifnγ, Il-1β, α-Sma* and *Tgfβ* in kidneys from the curcumin treatment and control groups examined by real-time quantitative PCR. (**D**) Representative images of kidneys by H&E staining and histological scores of renal lesions (scale bar: 50 μm). (**E**) Representative images of kidneys by Masson staining (scale bar: 50 μm). (**F**) Representative renal immunofluorescence staining showing IgG (green) and C3 (red) deposition in the curcumin and vehicle groups. Nuclei are stained with Hoechst (blue) (scale bar: 50 μm). The data are presented as the mean±SEM (n=6). ns, p˃0.05, *P<0.05, **p<0.01, ****p<0.0001.

To investigate the effect of curcumin on renal injury, we collected urine from the mice to measure their urine protein concentration. Compared with the vehicle group, treatment with curcumin significantly decreased the urinary protein concentration in MRL/lpr mice 9 days after the administration of curcumin (figure 2A). Consistently, treatment with curcumin significantly decreased the level of serum creatinine compared to that in the vehicle group (figure 2B). In addition, curcumin suppressed the expression of inflammatory *factors (Tnfα, Ifn-γ and Il-*1β) and fibrosis-related regulators (*α-Sma and Tgfβ*) in the kidney, as detected by RT-qPCR (figure 2C), which is consistent with the H&E staining results used to evaluate renal pathological inflammatory features. Compared with those of the vehicle group, the glomerulonephritis, interstitial nephritis and infiltration of lymphocytes surrounding blood vessels were significantly improved by treatment with curcumin, as evidenced by lower histological scores (figure 2D). The results from Masson staining also showed that curcumin effectively ameliorated renal fibrosis (figure 2E). Additionally, immunofluorescence was used to assess the deposition of immune complexes, including IgG and C3, in the glomeruli of MRL/lpr mice, and the results indicated that IgG and C3 deposition in the glomeruli was markedly reduced in the curcumin group (figure 2F). These results demonstrated that curcumin reduced renal injury and improved renal function in MRL/lpr mice with LN.

### Effects of curcumin on lupus nephritis in an alternative R848-induced lupus mouse model

**Figure 3:**
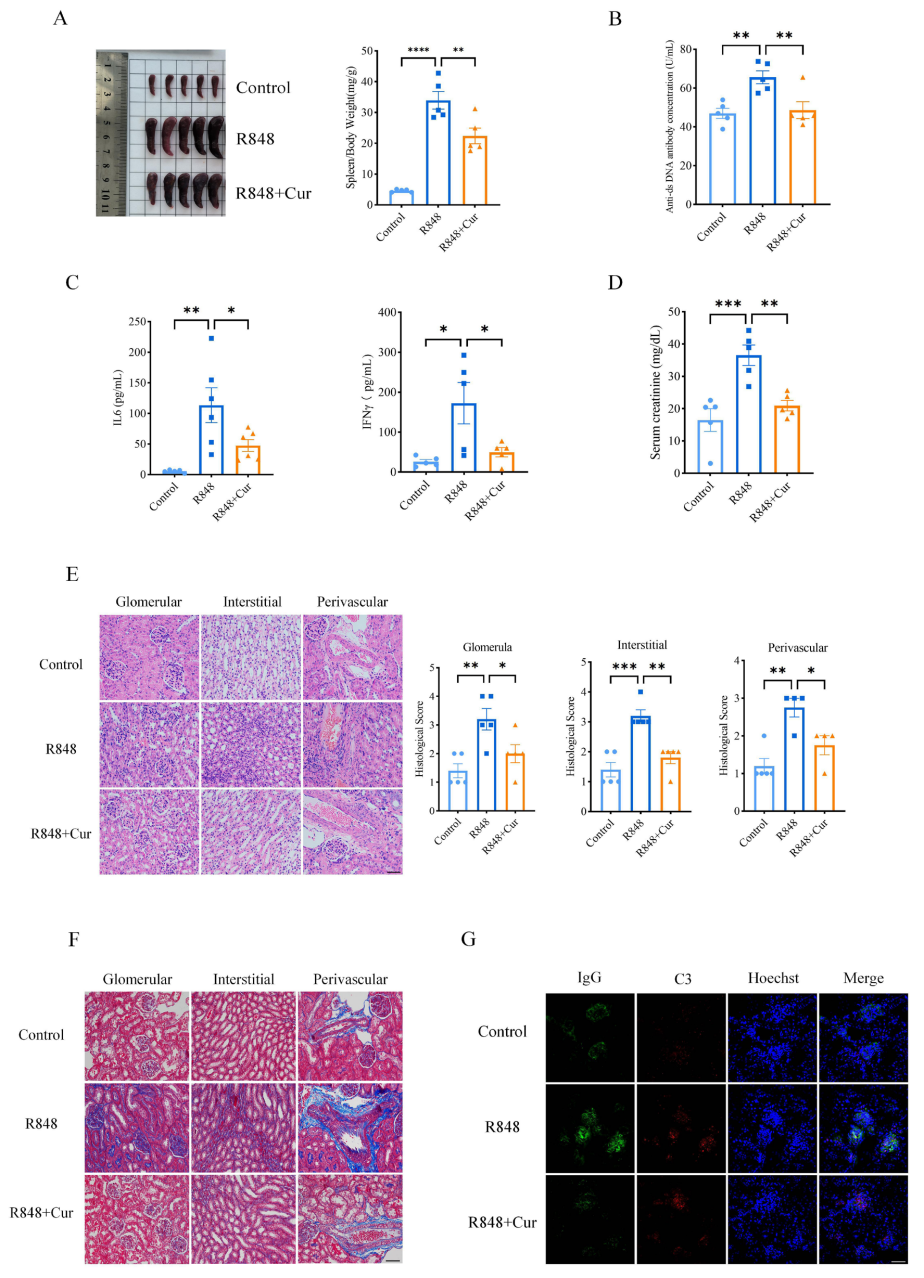
Curcumin ameliorated renal damage induced by R848. (**A**) Image of spleens and the spleen/body weight ratio (mg/g) of C57BL/6J mice from the control, R848 and R848 with curcumin treatment (R848+Cur) groups. (**B**) Serum anti-dsDNA antibody levels measured by ELISA in the control, R848 and R848 with curcumin treatment groups. (**C**) Serum levels of IL-6 and IFN-γ in the control, R848-treated and R848-treated groups were measured by ELISA. (**D**) Serum levels of creatinine in the control, R848-treated and R848-treated groups were measured by ELISA. (**E**) Representative images of kidneys by H&E and histological scores of renal lesions (scale bar: 50 μm). (**F**) Representative images of kidneys by Masson staining (scale bar: 50 μm). (**G**) Representative renal immunofluorescence staining showing IgG (green) and C3 (red) deposition in the control, R848 and R848 with curcumin treatment groups. Nuclei are stained with Hoechst (blue) (scale bar: 50 μm). The data are presented as the mean±SEM (n=5). *P<0.05, **p<0.01, ***p<0.001, ****p<0.0001. ds, double-stranded; IFN, interferon; IL, interleukin.

To further verify the therapeutic effects of curcumin on LN, we established a second lupus mouse model using the TLR-7/8 agonist R848. The mice received daily intraperitoneal injections of curcumin or vehicle. Treatment with curcumin significantly suppressed the increase in the spleen area induced by R848 (figure 3A). In addition, although the serum levels of anti-dsDNA antibody, creatinine, IL-6 and IFN-γ increased following R848 stimulation, curcumin treatment successfully reduced all of these levels (figure 3B–D). Furthermore, the characteristics of LN, including diffuse glomerular and glomerular basement membrane thickening, an increase in the mesangial matrix, interstitial mononuclear cell infiltration, tubular cast deposition and immune complex deposition, were also shown in the R848 model. However, renal pathological features were improved by treatment with curcumin in R848-induced lupus mice, with decreased histological scores compared to those of the R848 group without curcumin treatment (figure 3E). Masson staining further showed that R848 induced renal fibrosis, while curcumin ameliorated renal symptoms in the R848+curcumin group (figure 3F). Moreover, the deposition of immune complexes, such as IgG and C3, was also reduced in the curcumin group compared to the vehicle group (figure 3G). Taken together, our data suggested that systemic autoimmunity with local LN in mice induced by R848 was significantly improved by curcumin treatment, as was the case in MRL/lpr mice.

### Curcumin inhibits neutrophil infiltration and reduces inflammatory factors in lupus nephritis

**Figure 4:**
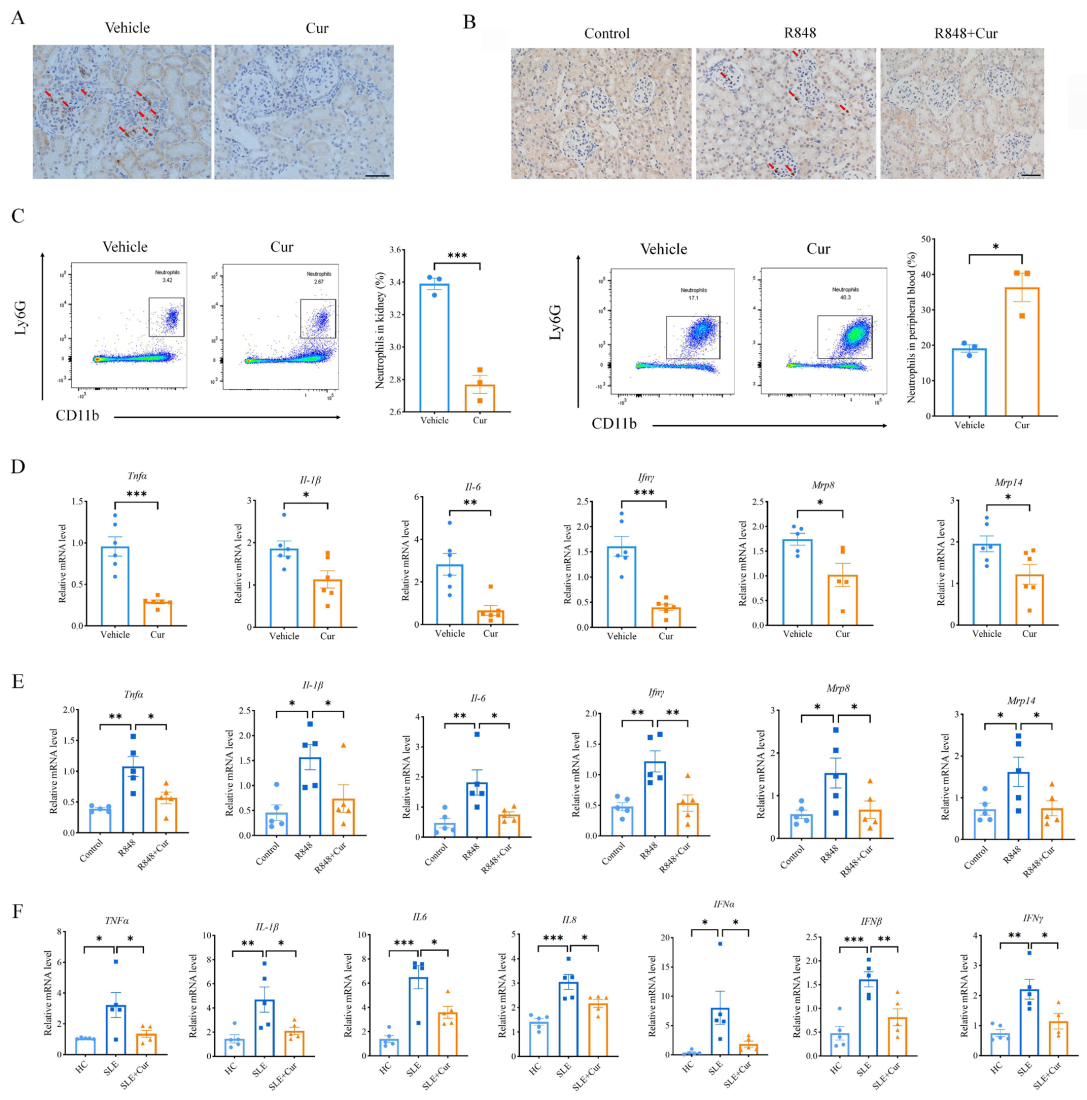
Curcumin inhibited renal infiltration and the production of inflammatory cytokines by neutrophils in LN. (**A**) Immunohistochemical staining of Ly6G in the kidneys of MRL/lpr mice treated with or without curcumin (scale bar: 50 μm). (**B**) Immunohistochemical staining of Ly6G in kidneys from C57BL/6J mice in the control, R848 and R848+curcumin treatment groups (scale bar: 50 μm). (**C**) Flow cytometric analysis of neutrophils in the kidney and peripheral blood of MRL/lpr mice treated with or without curcumin (n=3). (**D**) RT-qPCR detection of the mRNA expression of *Tnfα, Ifnγ, Il-1β, Il-6, Mrp8 and Mrp14* in the neutrophils of blood from MRL/lpr mice treated with or without curcumin (n=6). (**E**) RT-qPCR detection of the mRNA expression of *Tnfα, Ifnγ, Il-1β, Il-6, Mrp8 and Mrp14* in neutrophils in blood from C57BL/6J mice in the control, R848 and R848+curcumin treatment groups (n=5). (**F**) RT-qPCR detection of the mRNA expression of *TNFα, IFNα, IFNβ, IFNγ, IL-1β, IL-6 and IL-8* in neutrophils from the blood of patients with LN (n=5). The data are presented as the mean±SEM. *P<0.05, **p<0.01, ***p<0.001. IFN, interferon; IL, interleukin; mRNA, messenger RNA; TNF, tumour necrosis factor.

As the kidney is susceptible to inflammation and damage mediated by neutrophils in LN, as previously described,^16^ we further examined the infiltration of neutrophils in the kidneys to determine whether curcumin exerts its beneficial effects via neutrophils. Immunohistochemical identification of Ly6G revealed that the glomerulus in the kidney was infiltrated by neutrophils in both MRL/lpr-induced and R848-induced lupus mice, while treatment with curcumin indeed inhibited the recruitment of neutrophils to the glomerulus in both lupus mouse models (figure 4A–B). Moreover, flow cytometry analysis revealed a significant reduction in the percentage of CD11b^+^Ly6G^+^ neutrophils within the kidneys of MRL/Lpr mice following curcumin treatment, accompanied by a marked increase in the proportion of neutrophils in the peripheral blood (figure 4C).

To further clarify the effect of curcumin on neutrophils, we examined the expression of inflammatory factors in neutrophils extracted from the blood of MRL/lpr-treated and R848-treated mice. The RT-qPCR results showed that the mRNA levels of *Tnfα, Ifn-γ, Il-1β* and *Il-6* were significantly decreased after treatment with curcumin. Moreover, the expression of myeloid-related protein 8/14 (*Mrp8/14*), two cytokines that play a prominent role in regulating the production of proinflammatory factors and inducing neutrophil chemotaxis, was also significantly reduced by curcumin (figure 4D–E). Next, we collected neutrophils from patients with LN and incubated them with curcumin for 24 hours to determine whether curcumin can also have the same beneficial effects, as illustrated in the mouse models. As expected, the RT-qPCR results confirmed that the mRNA levels of inflammatory factors, including *TNFα, IFN-α, IFN-β, IFN-γ, IL-1β, IL-6* and *IL-8,* were significantly decreased with curcumin incubation (figure 4F). Taken together, these results demonstrated that curcumin inhibited the recruitment of neutrophils to the kidneys of patients with lupus and reduced the release of inflammatory factors from neutrophils.

### Curcumin inhibits neutrophil recruitment by regulating the PI3K/AKT/NF-κB signalling pathway

**Figure 5:**
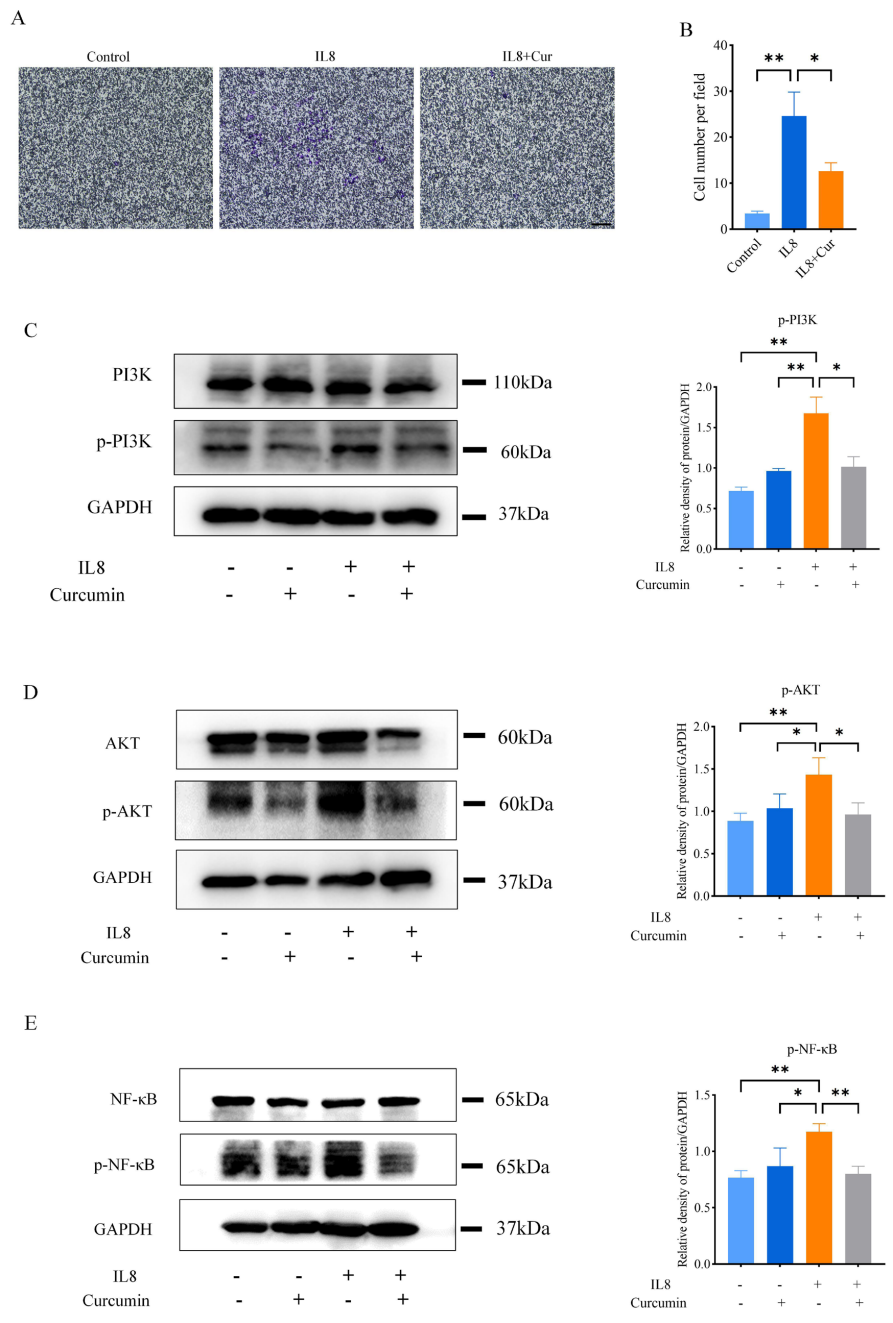
Curcumin reduced neutrophil recruitment by regulating PI3K/AKT/NF-κB pathway. (**A**) Representative images of neutrophil migration in the control, IL8 and IL8+curcumin groups (scale bar: 50 μm). (**B**) Statistical analysis of the numbers of migrating HL60 cells in the control, IL8 and IL8+curcumin groups from five random fields. (**C–E**) Representative western blot analysis results for p-PI3K, p-AKT and p-NF-κB in HL60 cells treated with or without IL8 and curcumin and quantification of the western blot analysis data. The data are presented as the mean±SEM. *P<0.05, **p<0.01. All experiments were repeated three times. NF-κB, nuclear factor-κB; p, phosphorylated; PI3K, phosphatidylinositol 3-kinase.

To explore the underlying molecular mechanism of the effect of curcumin on neutrophils, a cell migration assay with transwell chambers was performed using the human leucocyte cell line HL60. The results showed that the migration ability of HL60 cells was significantly increased under induction with IL-8, while treatment with curcumin markedly inhibited their migration ability (figure 5A–B). As neutrophils in the peripheral circulation respond to the induction of chemokines by activating several complex intracellular signalling pathways, including the PI3K and MAPK signalling pathways, and are subsequently recruited to inflammatory sites,^34^,^36^ we investigated the effect of curcumin on the PI3K/AKT signalling pathways in HL60 cells by western blot analysis. As shown in figure 5C and D, the levels of p-PI3K and p-AKT were significantly increased by stimulation with IL8 in HL60 cells but were significantly decreased by curcumin treatment. Moreover, the PI3K/AKT pathway downstream of NF-κB was further activated and phosphorylated upon p-PI3K and p-AKT activation, while curcumin inhibited the expression of p-NF-κB accordingly, as expected (figure 5E). Taken together, these results suggest that curcumin inhibits neutrophil recruitment and the subsequent release of inflammatory factors by regulating the PI3K/AKT/NF-κB signalling pathway.

## Discussion

In this study, we used both MRL/lpr mice and R848-treated mice to evaluate the therapeutic effect of curcumin and found that it effectively alleviated LN in both lupus models. Mechanistically, this improvement seemed to be achieved by inhibiting neutrophil migration and the release of inflammatory factors through the regulation of the PI3K/AKT/NF-κB signalling pathways.

In addition to playing an important role in host defence against microorganisms via phagocytosis, neutrophils can cause inflammation and tissue damage when they are abnormally activated by cytokines, chemokines and autoantibodies in inflammatory diseases.^37^ Neutrophils in autoimmune diseases direct inflammatory responses in a variety of ways, including secreting cytokines and chemokines to induce innate and adaptive immune responses, releasing receptors such as the IL-6 receptor to initiate trans-signalling and generating neutrophil extracellular traps and reactive oxygen species.^38^,^40^ Neutrophil may be a pathogenic factor for antibody-mediated LN.^16^ The infiltration of neutrophils in the kidney mainly occurs in the glomerulus, and subsequently, various enzymes released by neutrophils destroy the glomerular structure, including neutrophil elastase, myeloperoxidase and various cathepsins. In this study, we systematically confirmed the conspicuous infiltration of neutrophils within the renal tissue in both lupus mouse models, accompanied by a substantial release of inflammatory mediators, which collectively propel the initiation and exacerbation of LN. This finding provides an improved understanding of the role played by neutrophils in LN, which might generate opportunities for diagnostics and therapeutic interventions for LN.

Owing to its remarkable anti-inflammatory traits and safety profile, curcumin has been extensively used for the treatment of numerous ailments, including inflammatory bowel disease, arthritis, pancreatitis and cancer.^41^ Here, we administered curcumin intraperitoneally to the MRL/lpr and R848-induced lupus mice and found that it had a beneficial impact on LN through modulating the immune response via distinct immune cells and various cytokines. Particularly, a significant increase in Treg cell proportions was detected in MRL/lpr- and R848-challenged mice after curcumin treatment, suggesting that curcumin ameliorates LN by regulating immune responses. Moreover, curcumin has been shown to suppress neutrophil chemotaxis in kidneys to alleviate LN, as in inflammatory bowel disease.^42^ Complementarily, in vitro experiments in which neutrophils from patients with LN were cultured with curcumin effectively inhibited neutrophil activation under lupus-like conditions, a mechanism potentially mediated via modulation of the PI3K/AKT/NF-κB signalling pathway. Overall, our work elucidates the promotional effect of neutrophil-mediated inflammation in LN and identifies curcumin as a novel agent for LN treatment, highlighting its potential for regulating immune cell functions and mitigating inflammatory processes.

Despite curcumin demonstrating potential in the treatment of LN due to its anti-inflammatory and antioxidant properties and preliminary studies confirming its ability to inhibit neutrophil migration through the regulation of the AKT/NF-κB pathway—thereby positively intervening in the disease—its transition to widespread clinical use still faces significant hurdles. The foremost and most well-known limitation stems from the low bioavailability of curcumin.^43^,^45^ Despite exhibiting potent bioactivity in vitro, its efficacy inside the human body is compromised due to poor water solubility, rapid metabolism and limited intestinal absorption, often resulting in subtherapeutic levels in vivo. Addressing this issue necessitates breakthroughs in biotechnology, whether through chemical modifications to enhance its physicochemical properties or by employing nanotechnology and drug delivery systems such as liposomes and microemulsions aimed at increasing stability in the bloodstream and efficiency of tissue distribution, reducing first-pass effects and enhancing therapeutic potency. Therefore, future studies will focus on developing more efficacious curcumin derivatives or designing intelligent nanocarriers that circumvent the obstacles of low bioavailability, enhance the stability and targeting of these compounds in vivo, and thus facilitate precision therapy. On this basis, the meticulous design of curcumin derivatives could maximise therapeutic effects and reduce dose-dependent side effects, ensuring the safety and tolerability of the therapy.

Moreover, as LN is a complex autoimmune disease with an aetiology involving a combination of genetic factors, immune dysregulation and environmental triggers, disease manifestations significantly vary among individuals. The therapeutic efficacy of curcumin is substantially influenced by this intrinsic heterogeneity; a singular treatment approach fails to meet the needs of all patients. Therefore, when applying curcumin in treatment strategies, a comprehensive evaluation of patients is needed, considering their genetic background, disease stage and comorbidities, to explore personalised dosing regimens. This also implies that future research must focus more on precision medicine to more accurately predict which patients will benefit most from curcumin therapy. Overall, the role of curcumin in the treatment of LN should not be understood in isolation but rather as part of a multimodal treatment system. Future efforts will be dedicated to exploring how it can synergise with other immunomodulators, cytokine inhibitors or even emerging biological therapies to achieve complementary or enhanced therapeutic effects. Through meticulously designed combination therapies, it is possible to ensure treatment efficacy while reducing dependency on a single drug, lowering the risks of side effects or resistance associated with long-term treatment, and offering new therapeutic hope for patients who respond poorly to conventional treatments.

## Conclusion

This study showed that curcumin significantly alleviated pathological changes in both MRL/lpr- and R848-induced lupus model mice. The results also confirmed the regulatory effect of curcumin on neutrophil chemotaxis via the PI3K/AKT/NF-κB pathway. Hence, curcumin may be an effective Chinese medicine for the treatment of LN.

## Data Availability

No data are available.
